# Persistent Thalamic Sound Processing Despite Profound Cochlear Denervation

**DOI:** 10.3389/fncir.2016.00072

**Published:** 2016-08-31

**Authors:** Anna R. Chambers, Juan J. Salazar, Daniel B. Polley

**Affiliations:** ^1^Eaton-Peabody Laboratories, Massachusetts Eye and Ear InfirmaryBoston, MA, USA; ^2^Department of Biology, École Normale Supérieure, PSL Research UniversityParis, France; ^3^Department of Otolaryngology, Harvard Medical SchoolBoston, MA, USA

**Keywords:** medial geniculate body, hearing loss, homeostatic plasticity, compensatory plasticity, cochlear neuropathy

## Abstract

Neurons at higher stages of sensory processing can partially compensate for a sudden drop in peripheral input through a homeostatic plasticity process that increases the gain on weak afferent inputs. Even after a profound unilateral auditory neuropathy where >95% of afferent synapses between auditory nerve fibers and inner hair cells have been eliminated with ouabain, central gain can restore cortical processing and perceptual detection of basic sounds delivered to the denervated ear. In this model of profound auditory neuropathy, auditory cortex (ACtx) processing and perception recover despite the absence of an auditory brainstem response (ABR) or brainstem acoustic reflexes, and only a partial recovery of sound processing at the level of the inferior colliculus (IC), an auditory midbrain nucleus. In this study, we induced a profound cochlear neuropathy with ouabain and asked whether central gain enabled a compensatory plasticity in the auditory thalamus comparable to the full recovery of function previously observed in the ACtx, the partial recovery observed in the IC, or something different entirely. Unilateral ouabain treatment in adult mice effectively eliminated the ABR, yet robust sound-evoked activity persisted in a minority of units recorded from the contralateral medial geniculate body (MGB) of awake mice. Sound driven MGB units could decode moderate and high-intensity sounds with accuracies comparable to sham-treated control mice, but low-intensity classification was near chance. Pure tone receptive fields and synchronization to broadband pulse trains also persisted, albeit with significantly reduced quality and precision, respectively. MGB decoding of temporally modulated pulse trains and speech tokens were both greatly impaired in ouabain-treated mice. Taken together, the absence of an ABR belied a persistent auditory processing at the level of the MGB that was likely enabled through increased central gain. Compensatory plasticity at the level of the auditory thalamus was less robust overall than previous observations in cortex or midbrain. Hierarchical differences in compensatory plasticity following sensorineural hearing loss may reflect differences in GABA circuit organization within the MGB, as compared to the ACtx or IC.

## Introduction

Perception of environmental stimuli arises from the spatiotemporal patterning of spiking at higher stages of sensory processing (Logothetis and Schall, 1989; DeAngelis et al., 1998; Romo et al., 1998). Manipulating the balance of excitation and inhibition in auditory cortex (ACtx) changes sound-evoked cortical spiking patterns and imparts an immediate and specific effect on sound perception (Penfield and Perot, 1963; Letzkus et al., 2011; Froemke et al., 2012). Following cochlear hearing loss, the balance of excitation and inhibition tips toward hyperexcitability throughout the central auditory neuroaxis, increasing the “central gain” on afferent signals so as to partially compensate for a diminished input from the auditory periphery. Central gain cannot fully compensate for the loss of cochlear processing. To the contrary, by increasing spontaneous rates and correlated activity while decreasing the dynamic range of central auditory coding, excess central gain can further distort the representation of environmental sounds and even induce the perception of phantom sounds, contributing to pathophysiological processes such as hyperacusis and tinnitus, respectively (Roberts et al., 2010; Auerbach et al., 2014; Eggermont, 2016). Thus, dynamic central gain at higher stations of auditory processing may contribute in significant ways to sound perception in both normal and pathological conditions, though the underlying mechanisms and precise linkage to perception have yet to be fully revealed.

Many forms of hearing loss arise from an amalgam of cochlear hair cell dysfunction, loss of auditory nerve afferent synapses, and dysfunction of non-sensory cells that establish the endocochlear potential (Dubno et al., 2013). When characterizing disordered central sound representations and perception, it is challenging to disambiguate the contributions of abnormal transduction at the periphery from central plasticity. Importantly, some forms of hearing loss arise from a “pure” loss of Type-I spiral ganglion neurons (cochlear neuropathy) and/or their synapses with inner hair cells (cochlear synaptopathy), without hair cell loss (Kujawa and Liberman, 2009; Sergeyenko et al., 2013). In these cases, threshold sensitivity and cochlear amplification are normal, but afferent signals reaching the brain from the auditory nerve are greatly reduced and disordered. Patients with specific and extreme disruptions of auditory nerve afferent activity are diagnosed on the auditory neuropathy spectrum (Starr et al., 1996; Moser and Starr, 2016). Auditory neuropathy patients can present in the clinic with normal otoacoustic emissions, pure tone audibility and sound-evoked cortical potentials, yet have a near-complete loss of the auditory brainstem response (ABR) and acoustic reflexes in addition to great difficulty in psychophysical tasks that emphasize discrimination of temporal features, speech or sound location (Kraus et al., 2000; Zeng et al., 2005a).

Animal models of auditory neuropathy can be generated by cochlear round window application of drugs that selectively lesion inner hair cells or eliminate Type-I spiral ganglion neurons (Harrison, 1998). Due to the specificity of these drugs, one can disambiguate the contributions of central gain from peripheral pathology because afferent activity transmitted from the ear to the brain is substantially reduced without affecting cochlear transduction or amplification. Recordings from the central pathway in chinchillas treated with carboplatnin, a chemotherapy drug that selectively lesions inner hair cells, reveal a persistent loss of sound-evoked activity in the cochlear nucleus, a partial compensation for reduced peripheral input at the level of the inferior colliculus (IC) and a paradoxical increase in sound-evoked activity at the level of the ACtx (Wake et al., 1996; Qiu et al., 2000; Lobarinas et al., 2013). In a recent study, we induced a 95% loss of afferent synapses between Type-I spiral ganglion neurons and inner hair cells by applying ouabain to the cochlear round window and demonstrated that mice exhibit all of the defining features of auditory neuropathy described above in human patients: (1) normal otoacoustic emissions; (2) normal behavioral detection of pure tones; (3) normal sound-evoked cortical activity; (4) a near-complete loss of acoustic reflexes and the ABR; and (5) profound disruptions in the neural coding of speech tokens and temporally modulated sounds (Chambers et al., 2016). Our neurophysiological recordings lead us to conclude that central gain was increased after cochlear denervation, engaging a recovery of function in ACtx that was more complete than in the IC, but was limited to sound features that could be encoded by variations in overall firing rate, and not precise spike timing.

These observations motivated us to ask whether plasticity at the level of the thalamus more closely resembled the supra-normal responsiveness we observed in ACtx, the comparatively limited recovery in IC, or something different altogether. Although adjustments in central gain following hearing loss in the medial geniculate body (MGB) have received less attention than midbrain or cortical plasticity, sensorineural hearing loss has been associated with changes in thalamic GABA tone (Sametsky et al., 2015), physiological hyperactivity (Zhang et al., 2003; Chen et al., 2013, 2015; Kalappa et al., 2014) and behavioral hypersensitivity (Gerken, 1979). In the present study, we revisit our mouse model for profound unilateral cochlear neuropathy by directly comparing the loss of ABR to the preservation of auditory processing measured from thalamic units in awake mice.

## Materials and Methods

### Animals and Cochlear Denervation

All procedures were approved by the Animal Care and Use Committee of the Massachusetts Eye and Ear Infirmary and followed guidelines established by the NIH for the care and use of laboratory animals. Selective degeneration of Type-I spiral ganglion neurons was achieved by applying a 1 mM solution of ouabain octahydrate (Sigma) and sterile water to the left round window niche. The sham condition entailed applying only sterile water to the exposed round window niche. Detailed protocols for round window ouabain application have been published previously (Lang et al., 2005; Yuan et al., 2014). Animals were anesthetized with ketamine (120 mg/kg) and xylazine (12 mg/kg), with half the initial ketamine dose given when required. The connective tissue, underlying muscle and the facial nerve were blunt dissected and held away from the bulla with retractors. A small opening was made in the bulla with the tip of a 28.5-gauge needle. The exposed round window niche was filled with 1–2 μL of the ouabain solution using a blunted needle. Ouabain was reapplied five more times at 15-min intervals, wicking the existing solution away before each application. Preliminary measurements of the ABR and distortion product otoacoustic emissions (DPOAE) were made after the sixth application to confirm an immediate ABR threshold shift without any change in otoacoustic emission thresholds or amplitudes (see below for cochlear function testing procedures). Additional rounds of ouabain solution were applied, as necessary, until the ABR threshold at 16 kHz was 55–70 dB SPL (for ouabain plus sterile water) or remained at control levels (25–35 dB SPL, for sterile water alone). The incision was sutured and the mouse was given Buprenex as an analgesic before being transferred to a warm recovery cage (0.5 mg/kg). Of the 21 mice that underwent the surgery for cochlear round window exposure, 12 met our criteria for the preservation of otoacoustic emissions with the targeted levels of ABR threshold elevation, of which six provided high quality MGB unit recordings in head-fixed mice 1 month later. Of the four sham-treated animals that participated in cochlear function measurements, two provided high quality MGB recordings under head fixation 1 month after treatment.

### Cochlear Function Testing

ABR measurements were performed with transdermal electrodes under ketamine/xylazine anesthesia with core body temperature maintained at 36.5°C with a homeothermic heating pad. ABR stimuli were tone pips, 5 ms in duration (8, 16 and 32 kHz, from 20 to 80 dB SPL in 5 dB steps, 0.5 ms raised cosine onset and offset ramps). ABR wave 1b was identified manually and amplitude calculations were performed with custom software (LabView). The threshold for ABR was defined as the lowest stimulus level at which a repeatable waveform morphology could be visually identified. Visual identification of the waveform was validated with a semi-automated algorithm that identifies peaks and troughs of putative ABR waves by first calculating the negative zero crossings (NZCs) of the first derivative of the recorded waveform. To avoid mislabeling peaks in noisy signals, the algorithm eliminates spurious peaks by setting a threshold for NZC amplitude based on the noise floor, calculated from the standard deviation of the first 1 ms of the signal (Buran et al., 2010). The 2f1-f2 distortion DPOAE was measured in the ear canal using primary tones with a frequency ratio of 1.2 and level difference of 10 dB, incremented in 5 dB steps from 20 to 80 dB SPL.

### Preparation for Head-Fixed Unit Recordings

Several days before unit recordings, the periosteum was removed and a titanium head plate was affixed to the dorsal surface of the skull overlying bregma using dental cement (C&B Metabond). On the day of recording, a small (~2 × 2 mm) craniotomy was made on the dorsal surface of the skull overlying the MGB (1 mm rostral to the lambdoid suture, 2–3 mm lateral to the midline). A chamber was built around the craniotomy with UV-cured cement (Kerr Optibond) and filled with antibiotic ointment. Upon completion of unit recording for the day, the animal was briefly anesthetized with isoflurane (5% induction, 1.5% maintenance) and the chamber was sealed with a cap of UV-cured cement.

On the day of recording, the head was immobilized by attaching each prong of the head plate to a rigid clamp (Altechna). The animal’s body rested atop a rotating disk, coated with a sound-attenuating polymer that was mounted on a low-friction, silent rotor (Zhou et al., 2014). We continuously monitored the eyelid and status of the rotating disk to confirm that all recordings were made in the awake condition. To make recordings from the MGB, a 16-channel silicon probe (177 μm^2^ contact area, 50 μm between contacts, NeuroNexus) was inserted into the MGB with a dorsal approach using a hydraulic microdrive (FHC). To identify the ventral subdivision of the MGB (MGBv), we first recorded lateral to the MGB, in the hippocampus, and then marched the electrode medially in 0.1 mm steps until we had at least eight contiguous channels with broadband noise-evoked spiking activity. In so doing, we were assured of recording from the lateral bank of the MGB, which contains the MGBv and, depending on the caudal-rostral coordinates, might also contain recording sites in the dorsal subdivision and suprageniculate nucleus (Anderson and Linden, 2011; Hackett et al., 2011). The recording locations obtained via the above approach were verified in a series of pilot experiments using post-mortem reconstruction of electrolytic lesion locations (data not shown).

### Neurophysiology Data Collection

Raw signals were digitized at 32-bit, 24.4 kHz (RZ5 BioAmp Processor; Tucker-Davis Technologies, Alachua, FL, USA) and stored in binary format. Subsequent analyses were performed in MATLAB (MathWorks). The signals were notch filtered at 60 Hz and then bandpass filtered at 300–5000 Hz with a fifth-order acausal Butterworth filter. Multiunit spiking activity was limited to spike waveforms that were at least 3.5 standard deviations above the mean of a 10 s running average for each electrode (OpenEx, Tucker-Davis Technologies, Alachua, FL, USA). We employed an online automated algorithm to remove the common mode signal from the multiunit spike record. Factors such as line noise and movement artifacts contribute to the common mode signal, which appears simultaneously and in-phase across all recording channels. The common mode signal is calculated by averaging across all recording channels, and the rejection is achieved by subtracting the averaged signal from each individual channel (Bierer and Anderson, 1999).

### Stimuli

Stimuli were generated with a 24-bit digital-to-analog converter (National Instruments model PXI-4461). For DPOAE and ABR tests, as well as during surgeries, stimuli were presented via in-ear acoustic assemblies consisting of two miniature dynamic earphones (CUI CDMG15008–03A) and an electret condenser microphone (Knowles FG-23339-PO7) coupled to a probe tube. Stimuli were calibrated at the tympanic membrane in each mouse before recording. For awake recordings, stimuli were presented via a free-field electrostatic speaker (Tucker-Davis Technologies, Alachua, FL, USA) placed approximately 10 cm from the left ear canal. Free-field stimuli were calibrated before recording using a wide-band ultrasonic, acoustic sensor (Knowles Acoustics, model SPM0204UD5).

Acoustic stimulation was restricted to the left (treated) ear. While mice were briefly anesthetized to remove the cap of the recording chamber, a dense foam earplug (3M), cut to fit the ear canal of a mouse (~2 mm diameter) was securely fit into the external auditory meatus of the right ear. We have previously confirmed that this unilateral earplug approach affords at least 60 dB of attenuation across the frequencies tested (Chambers et al., 2016). Responses recorded during the experiment could arise from the stimulation of remaining nerve fibers of the treated ear, or from inadvertent stimulation of the untreated ear, through the earplug, on the opposite side of the head (acoustic crosstalk). In order to minimize acoustic crosstalk, we set a global ceiling on stimulus levels of 80 dB SPL and excluded any recording sites with thresholds high enough so that acoustic cross-talk could not unambiguously be ruled out (above 65 dB SPL, <3% of recordings). A subset of mice were tested at the end of the experiment with earplugs in both ears, to confirm that no sound-evoked responses were observed. These combined controls ensured that all sound-evoked activity was mediated by the left, contralateral ear.

### Data Analysis

#### Frequency Response Areas, Rate-level Functions, Chirp Train Synchronization

Sound-driven sites were identified by binning the peristimulus time histogram (PSTH) at 10 ms resolution, and determining if at least one bin within the stimulus presentation window (starting at 0 ms re: presentation and ending 50 ms after stimulus offset) was at least 3 SDs above the spontaneous firing rate distribution. Frequency response areas (FRAs) were measured with pseudorandomly presented tone pips (50 ms duration, 4 ms raised cosine onset/offset ramps, 0.5–1 s intertrial interval) of variable frequency (4–64 kHz in 0.15 octave increments) and level (0–70 dB SPL in 5 dB increments). Each tone pip was repeated twice and responses were averaged.

D-prime values for tuning quality of the FRA were calculated as described previously (Guo et al., 2012). Briefly, the mean spike count for 30 frequency-level combinations were taken at random from inside vs. outside the boundaries of the FRA. This process was repeated 1000 times to create tone-evoked and tone-independent spiking distributions. The difference between the mean of the two distributions, divided by their arithmetic average SD, yielded the d-prime of the unit.

Rate-level functions were calculated from responses to broadband chirp stimuli. Responses were smoothed with a 3-point moving average and fit with a six parameter Gaussian function (Watkins and Barbour, 2010).

Frequency-modulated chirps were 1 ms in duration, and spanned 4–64 kHz. The FM rate was calculated to compensate for the mouse basilar membrane group delay in order to generate a synchronous, equipotent displacement of the cochlear partition (Spankovich et al., 2008). Vector strength (VS) of responses to chirp trains was calculated as follows (Yin et al., 2011):

(1) $$\text{VS}\;=\;\frac{\sqrt{{\left(\sum_{i\;=\;1}^{n}\cos\theta \right)}^{2}+{\left(\sum_{i\;=\;1}^{n}\sin\theta \right)}^{2}}}{n}$$

Where VS is the vector strength, *n* is the number of spikes over all trials, and θ is the phase of each spike in radians. Phase-projected vector strength (VSpp) is calculated as follows:

(2) $$\text{VSpp}\;=\;{\text{VS}}_{\text{t}}\cos\left(\varphi_{t}-\varphi_{c}\right)$$

Where VSpp is the phase-projected vector strength per trial, VS_t_ is VS per trial, and *φ_t_* and *φ_c_* are the trial-by trial and mean phase angle in radians. Cycle-by-cycle vector strength (VS_cc_), a metric that describes both the degree of synchronization and the reliability of the synchronization across the duration of the stimulus, is calculated similarly to VSpp, except that it is computed for each cycle individually rather than over the entire stimulus period. A VSpp value is generated per cycle, and VSpp values over all cycles are averaged together to generate the VScc.

#### Speech Stimuli

We made recordings of an adult female speaker (fundamental frequency ~250 Hz) while she produced 12 speech tokens (consonant-vowel-consonant) in a sound treated room (sampling rate = 192 kHz). The first consonant in each token was one of the six stop consonants (/b, d, g, p, t, k/), which can be categorized by their place of articulation (POA) and voice onset time (VOT). Stop consonants have one of three POAs, denoting where the obstruction in the vocal tract is made (e.g., at the lips for /p/, the alveolar ridge for /t/, and the glottis for /g/). POA change is accompanied by characteristic alterations of the dynamic resonances of the vocal tract. VOT denotes the delay between initiation of a stop consonant and vibration of the vocal folds. Stop consonants are categorized as either voiced (/b/, /d/, and /g/) by their short VOTs or unvoiced (/p/, /t/, and /k/) by their longer VOTs. The central vowel in each token was either /i/or /α/, which sound like “ee” and “ah” respectively. The consonant /d/ was always the final sound in each token. Therefore, any two tokens could differ by as many as three attributes (POA, VOT, and vowel) or as few as one. The TANDEM-STRAIGHT vocoder was used to resynthesize the natural vocalizations and then frequency shift them into the mouse hearing range (shift = 4 octaves) without distorting the spectrotemporal envelopes of the source material (Kawahara and Morise, 2011).

#### PSTH Classifier Model

The PSTH classifier model compares the Euclidean distance between the population single trial spike train elicited by a given stimulus to the response templates created for each stimulus (Foffani and Moxon, 2004). The spike train is classified as being generated in response to the stimulus from which its distance is minimal. A response window of 0.1 s was aligned with stimulus onset. A matrix with T × S rows and B × N columns was constructed, where T is the number of stimulus repeats (*n* = 20), S is the number of stimuli, B is the number of bins that contain spikes, and N is the number of recording sites in the ensemble (1–20 units).

Let *v*_i,j_ represent the spike count in *i*th row and *j*th column of the matrix, where *i* goes from 1 to ST and *j* goes from 1 to NB. Templates for each stimulus were defined as ${\bar{v}}^{s}$ = [${\bar{v}}_{1}^{s}$, … , ${\bar{v}}_{NB}^{s}$], where the *j*th element is calculated as

(3) $${\bar{v}}_{y}^{j}\;=\;\frac{1}{T}\sum_{i\in s}v_{ij}^{s}$$

For each trial *v^i^* = [*v_i,1_*, … ,*v_i,B_*], the Euclidean distance between that trial and each stimulus template ${\bar{v}}^{s}$ = [${\bar{v}}_{1}^{s}$, … , ${\bar{v}}_{B}^{s}$] was defined as

(4) $$d_{s}^{i}\;=\;\sqrt{\sum_{j\;=\;1}^{NB}{\left(v_{i}{,}_{j}-{\bar{v}}_{j}^{s}\right)}^{2}}$$

Using these distances, the spike train is classified as being generated by the stimulus class represented by the closest template, resulting in an outcome vector of *c* = [*c_1_*, … ,*c_ST_*], where

(5) $$c_{i}\;=\;\arg\min\left(d_{s}^{i}\right)$$

Therefore, *c* will be of the same length as ST^s^ (total number of trials for all stimuli), and each element *c*_i_ indicates to which stimulus the *i*th trial is assigned.

## Results

### Ouabain Induces Profound Spiral Ganglion Neuron Degeneration While Sparing Cochlear Amplification

**Figure 1:**
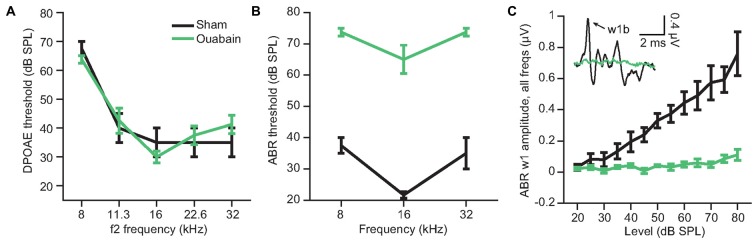
**Ouabain application to the cochlear round window induces profound denervation, while sparing cochlear amplification.** The loss of afferent activity in the auditory nerve was estimated from the amplitude and threshold of wave 1b of the auditory brainstem response (ABR) 30 days after cochlear ouabain treatment. Outer hair cell function, measured via distortion product otoacoustic emission (DPOAE) threshold, is left intact. **(A)** DPOAE threshold by f2 frequency, measured at 30 days after ouabain (green) or sham (black) treatment. **(B)** ABR threshold by tone frequency. **(C)** ABR wave 1b amplitude vs. level, averaged across three tone frequencies per animal (8, 16 and 32 kHz). Inset shows example ABR waveforms in the sham and ouabain-treated conditions (16 kHz, 70 dB SPL). Arrow indicates wave 1b. Data and error bars are the mean ± SEM.

We induced a profound afferent denervation of the cochlea by applying ouabain to the left cochlear round window. Ouabain is a Na^2+^/K^+^ ATP-ase pump inhibitor that, in gerbils and mice, selectively eliminates Type-I spiral ganglion neurons while sparing other types of afferent and efferent fibers in the auditory nerve as well as sensory and non-sensory cells of the inner ear (Lang et al., 2005). Previous studies of cochlear anatomy from our group indicate that ouabain eliminates approximately 95% of Type-I afferent synapses across all regions of the cochlear frequency map (Yuan et al., 2014; Chambers et al., 2016). One month after cochlear denervation, DPOAE thresholds were indistinguishable between the sham and ouabain-treated mice (Figure 1A, one-way analysis of variance (ANOVA), *F*_(1)_ = 1.6, *p* = 0.33; ouabain treated *n* = 6, sham treated *n* = 4), indicating that hair cell-dependent cochlear amplification was unaffected by ouabain. By contrast, thresholds for wave 1b of the ABR were elevated by an average of 38.2 dB after ouabain treatment (Figure 1B, ANOVA, *F*_(1)_ = 244.7, *p* < 0.0001), and wave 1b amplitudes were greatly attenuated (Figure 1C, ANOVA, *F*_(1)_ = 90.07, *p* = 0.0002), indicating a profound cochlear neuropathy.

### Despite Near-Complete Denervation, Some Sites in the MGB Show Robust Sound Driven Responsiveness 30 days After Ouabain Treatment

**Figure 2:**
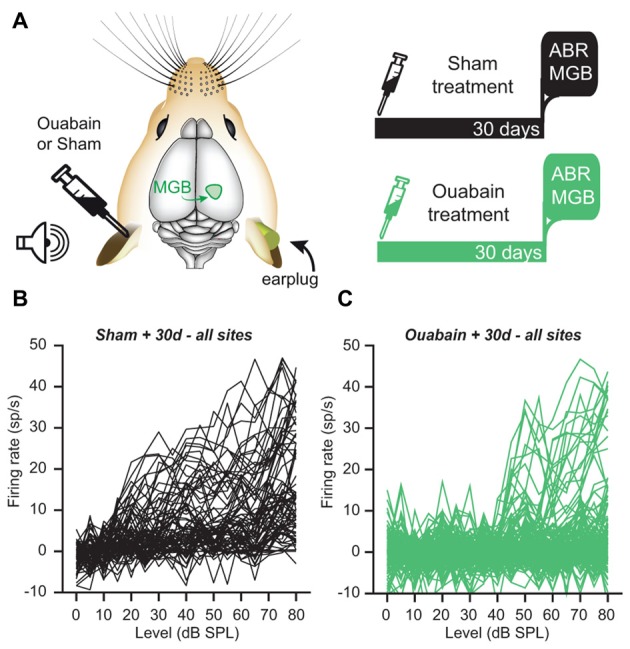
**Despite the loss of an ABR, a minority of recording sites in the medial geniculate body (MGB) of the thalamus show robust, sound-driven responses 30 days after ouabain treatment. (A)** Schematic of the experimental configuration and timeline. Animals were subjected to unilateral ouabain or sham (sterile water) treatment. Extracellular multi-unit recordings were made in the contralateral MGB of awake mice 30 days after treatment. During recording experiments, acoustic stimulation was delivered to the contralateral, treated ear while a dense foam earplug was secured in the ipsilateral, untreated ear. **(B,C)** Baseline-subtracted rate-level functions in response to broadband chirp stimuli for all MGB recording sites, whether significantly responsive or not. **(B)** Following sterile water application at the contralateral round window, most MGB multi-unit responses to broadband chirps of increasing level were robust and monotonically increased with stimulus intensity. **(C)** Following ouabain treatment, many MGB sites were unresponsive to acoustic stimuli, while others showed robust sound-driven activity with comparable peak amplitudes to the sham condition.

Our prior study demonstrated a persistence of central auditory processing and sound detection abilities in the face of profound cochlear denervation (Chambers et al., 2016). We determined that overall recovery of function was more extensive in the ACtx than the IC, and was more robust in both brain areas for sound features that could be encoded by overall variations in firing rate than for sound feature representations that were based on precise spike timing. To address whether the level and form of compensatory plasticity in the MBG was more comparable to the cortex or IC (or was in a category of its own), we recorded multiunit activity 30 days after ouabain or sham treatment from the right MGB of awake, head-fixed mice (Figure 2A). Acoustic stimuli were presented via a free-field speaker directed at the left ear. We inserted a dense foam earplug into the right, untreated ear prior to the start of unit recordings, which provided over 60 dB of attenuation across the range of frequencies used in our test stimuli and ensured that sound-evoked spiking was mediated by the left, treated ear (Chambers et al., 2016).

The MGBv was located using a combination of stereotactic coordinates and functional mapping (see “Materials and Methods” Section). Multiunit recordings from the MGB of sham-treated mice (*n* = 80 units) showed a high proportion of robustly sound-driven sites with monotonically increasing firing rates in response to broadband “chirp” stimuli of increasing intensity (Figure 2B). By contrast, MGB recordings from ouabain-treated mice (*n* = 160 units) revealed a substantial loss of sound-driven sites. However, we observed a minority of sites that were driven by sound with similar peak firing rates—despite higher thresholds—to those seen in the sham condition (Figure 2C), which would not have been expected from the virtually flat wave 1b ABR input/output functions (Figure 1C). In this respect, persistent MGB responses recorded 30 days after contralateral cochlear denervation were reminiscent of our previous description of compensatory plasticity in IC and ACtx.

### Rate-Level Functions in the MGB Show Evidence of Gain Enhancement in Ouabain Treated Animals, and Level Encoding is Partially Intact

**Figure 3:**
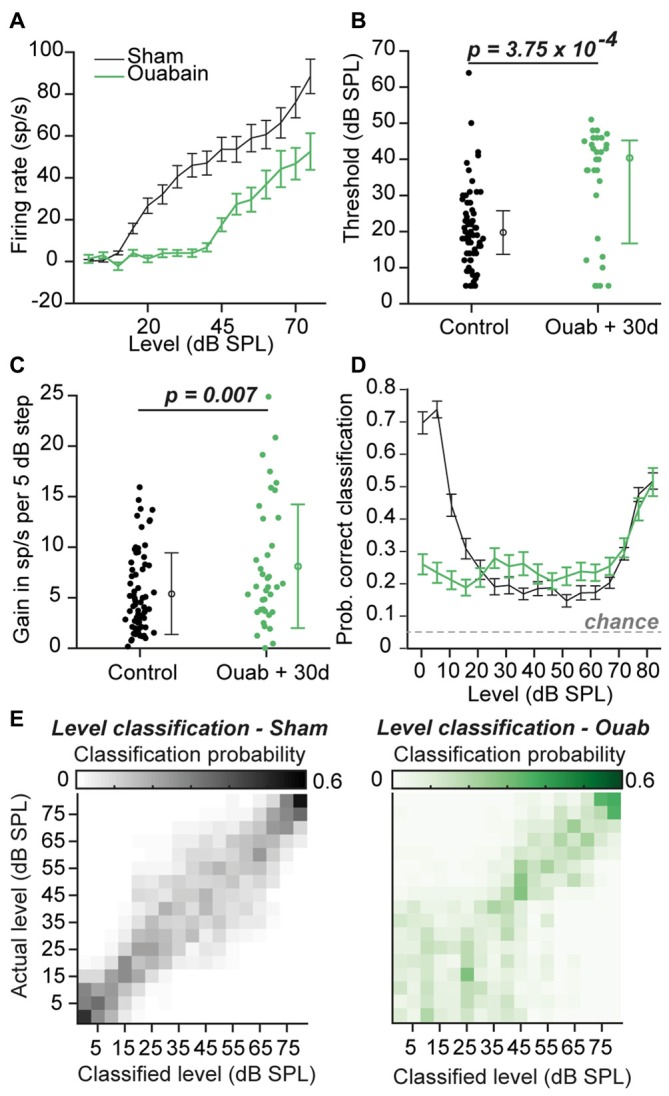
**Firing rate vs. sound intensity functions after ouabain treatment show elevated thresholds and enhanced gain. (A)** Average rate-level functions of all significantly responsive sites (see “Materials and Methods” Section) in the two experimental groups, in response to broadband chirp stimuli. Sp/s = spikes per second. Error bars are mean ± SEM. **(B)** Minimum thresholds are significantly elevated in the ouabain-treated group (*p* = 3.75 × 10^−4^; Wilcoxon rank sum test; horizontal lines = median). Error bars are median ± interquartile range. **(C)** The slope of the initial rising segment of rate level functions is significantly enhanced in the ouabain treated group, indicating increased gain (measured as the increase in spiking rate per 5 dB step, *p* = 0.007, unpaired *t*-test). Error bars are mean ± standard deviation. **(D)** Mean probability of correct classification by level in both groups, determined by a peristimulus time histogram (PSTH)-based Euclidean distance classifier (see “Materials and Methods” Section). Classification is considered “correct” within a ±5 dB range. The probability of assigning the correct sound level by chance is indicated by the dashed gray line. Error bars are mean ± SEM. **(E)** Confusion matrices of level classification in sham (left) and ouabain treated (right) groups, averaged across individual multi-unit sites, with PSTHs binned at 10 ms resolution.

To determine if the partial preservation of auditory processing after cochlear denervation was sufficient to encode basic features of auditory stimuli, we first investigated sound level coding among the sub-population of MGB recordings sites that was significantly driven by sound (Figure 3A). Although minimum response thresholds were significantly elevated after ouabain treatment (Figure 3B; *p* = 3.75 × 10^−4^, Wilcoxon rank-sum test), the increase in firing rate with increasing sound level was robust and offered a potential useful basis for encoding variations in sound intensity. In fact, sensory gain, measured as the increase in firing rate per unit increase in sound intensity, was significantly greater in ouabain-treated animals vs. sham-treated animals (Figure 3C; *p* = 0.007, unpaired *t*-test). To test whether the increased gain could offset the increased thresholds and number of unresponsive units to preserve a useful code for sound level classification, we utilized a PSTH-based Euclidean distance metric to classify neural responses (see “Materials and Methods” Section). The probability of accurate classification (veridical SPL ±5 dB) remained above chance across the full range of SPLs and was nearly identical between sham- and ouabain-treated recordings from 25 to 80 dB SPL (Figure 3D). Unsurprisingly, significant deficits in sound intensity coding were noted at low sound intensities in the ouabain-treated recordings on account of elevated thresholds. Average confusion matrices from the sham-treated (Figure 3E, left) and ouabain-treated recordings (Figure 3E, right) indicate that sound level classification errors could typically be attributed to neighboring or near-neighboring sound levels, underscoring that a neural code for rudimentary sound level classification can be identified in the MGB despite the absence of a measurable ABR.

### Pure Tone Frequency Representations in the MGB are Partially Recovered 30 Days After Ouabain Treatment

**Figure 4:**
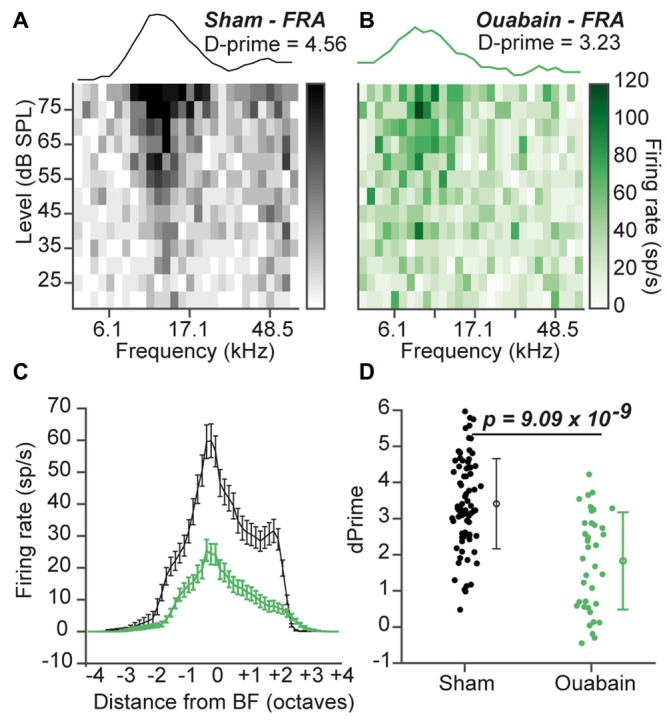
**Diminished pure tone receptive fields persist 30 days after ouabain treatment. (A,B)** Example frequency response area (FRA) from a single multiunit site from a sham **(A)** and ouabain-treated **(B)** mouse. Line plot above each FRA is the average tuning function across three stimulus levels, centered on the site’s best level. The FRA tuning quality is measured with the D-prime statistic (see “Materials and Methods” Section). **(C)** Mean frequency response functions from significantly responsive sites in sham and ouabain groups. Error bars are mean ± SEM. **(D)** D-prime values indicate a significant decrease in FRA quality 30 days after ouabain treatment (*p* = 9.09 × 10^−4^, unpaired *t*-test). Error bars are mean ± standard deviation.

In our previous study, normal tone frequency encoding in the ACtx of ouabain-treated mice was preserved even after only 1 week of recovery. Surprisingly, tone-driven responses at best level often exceeded average control amplitudes at 1 month, and no significant change in the overall quality of tone frequency tuning was observed in the ACtx between the ouabain-treated and control conditions (Chambers et al., 2016). To investigate whether this degree of compensatory recovery could also be observed in the MGB, we measured FRAs to pure tones of varying frequency level and analyzed all multi-unit sites that were significantly driven by sound (Figure 4). Example FRAs with tuning functions at best level ± 5 dB are shown in Figures 4A,B, where the tuning quality is quantified as d-prime, a metric that represents the separability of the firing rate distributions within vs. outside the receptive field boundary (see “Materials and Methods” Section). Although well-defined tonal receptive fields were observed in the MGB 30 days after contralateral denervation, FRAs showed reduced tone-evoked firing rates across the frequency response function (Figure 4C) and a significantly reduced overall tuning quality (Figure 4D; *p* = 9.09 × 10^−4^, unpaired *t*-test).

### The Encoding of Broadband Pulse Train Frequency is Significantly Impaired in MGB Units 30 Days After Ouabain Treatment

**Figure 5:**
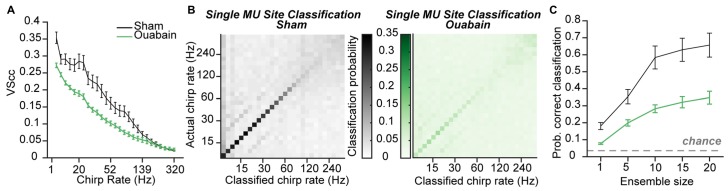
**Temporal coding persists, with impairment, after correcting for threshold shifts. (A)** Mean cycle-by-cycle vector strength (VScc) values from all significantly responsive sites, in response to chirp trains presented at 20 dB above the rate-level function threshold. **(B)** Confusion matrices for mean chirp rate classification using the Euclidean distance-based PSTH classifier on individual multi-unit sites with a 10 ms bin width from sham-treated (left) or ouabain-treated mice (right). **(C)** Mean probability of correct classification is impaired in the ouabain-treated group for ensemble sizes ranging from 1 to 20 multi-unit sites. The probability of assigning the correct broadband pulse rate by chance is indicated by the dashed gray line. Error bars are mean ± SEM.

Patients on the auditory neuropathy spectrum can present in the audiology clinic with only a mild or moderate hearing loss despite a near-absent ABR, yet invariably have profound temporal discrimination deficits (Starr et al., 1996; Zeng et al., 2005a). A critical observation that arose from our mouse model of auditory neuropathy was that increased central gain was sufficient to restore neural coding of sound level and frequency in the ACtx, yet temporal coding deficits showed comparatively little recovery in the IC (Chambers et al., 2016). Spike synchronization coding strategies that dominate in the midbrain and brainstem are largely reformatted to non-synchronized, rate-based representations at the level of the cortex, which limited our earlier analysis of temporal processing deficits after ouabain treatment to the IC (Bendor and Wang, 2007; Wang et al., 2008). Temporal coding in the MGB is a hybrid of synchronized and non-synchronized coding (Bartlett and Wang, 2006), which allowed us to investigate whether central gain was able to restore temporal synchronization in an auditory forebrain structure. To address this question, we analyzed the responses of sound-driven MGB multi-unit sites to broadband pulse trains (Figure 5). In order to correct for threshold shifts observed after ouabain treatment, pulse trains were presented 20 dB above the threshold measured from rate-level functions for each recording site.

Synchronization of the neural response to pulse train frequency was measured via VScc, a metric that describes both the timing and the reliability of the neural response to amplitude modulated stimuli (see “Materials and Methods” Section; Yin et al., 2011). In multiunit recordings made from the IC 30 days after ouabain, VScc was reduced by approximately 0.25 compared to controls (Chambers et al., 2016). Spike synchronization was significantly reduced in the MGB of ouabain-treated mice, but degradation of temporal coding was less extreme on average than what we observed in the IC (mean VS across pulse frequency 0.11 (ouabain) vs. 0.16 (sham), *p* = 0.0004, unpaired *t*-test, Figure 5A). By using the PSTH-based classifier to assess the accuracy of temporal coding using rate- or timing-based spike patterning, we previously found that optimal temporal decoding in the IC was achieved with single neurons and small bin widths in control conditions, but was more accurate when distributed over ensembles of 10–20 recording sites with coarser temporal binning after cochlear denervation. We therefore applied the PSTH-based classifier method on our thalamic recordings to ask whether a similar transition from single site to ensemble-based decoding occurred in the MGB and to test whether the stimulus pulse rate could be identified from the distribution of spikes over time (i.e., the PSTH), whether synchronized or not.

For both the sham and ouabain conditions, acoustic pulse rate could be optimally decoded with a temporal resolution of 10 ms (main effect for bin size *p* < 0.0001 for both ouabain and sham conditions, one-way ANOVA). Decoding the pulse rate of broadband chirp trains from single MGB recording sites at the optimal bin size revealed an overall loss of accuracy, particularly at higher pulse rates after cochlear denervation (Figure 5B). Mean classification accuracy across all pulse rates was improved by pooling across multiple recording sites in both the sham- and ouabain-treated cases (main effect for ensemble size *p* < 0.0001 for both ouabain and sham conditions, one-way ANOVA, Figure 5C), though the degree of improvement with increasing ensemble size was significantly more pronounced for the sham treated group compared to the ouabain treated group (interaction term, *F*_(4)_ = 13.63, *p* < 0.0001, mixed design ANOVA).

These findings reveal an intriguing pattern of differences in the preservation of temporal coding in the midbrain, thalamus and cortex after profound contralateral denervation. In ACtx, spike synchronization provides little information about temporal pulse rate in ouabain-treated or sham recordings, yet the weak overall temporal coding nearly recovered to control levels when measured with coarse temporal binning and large ensemble sizes. In IC, spike synchronization was greatly reduced after ouabain treatment, though information about the acoustic pulse rate was partially preserved with coarser temporal binning across larger ensembles of recording sites. In MGB, there was a significant but comparatively small loss of spike synchronization, yet pulse rate classification did not disproportionately benefit from pooling across larger ensembles of neurons.

### Speech Token Classification is Significantly Impaired in the MGB of Ouabain-Treated Animals

Individuals on the auditory neuropathy spectrum often report that they can hear speech, but not understand it (Starr et al., 1996; Kraus et al., 2000; Berlin et al., 2010). Because accurate speech reception in quiet backgrounds is primarily dependent on the proper encoding of temporal fluctuations in the sound pressure envelope (Shannon et al., 1995; Zeng et al., 2005b), and because subjects on the auditory neuropathy spectrum demonstrate profound temporal processing deficits despite a preservation of basic audibility, it is generally understood that the dichotomy between hearing speech and understanding speech reflects a broader failure of temporal processing (Zeng et al., 2005a). It is less clear to what extent deficits in the temporal processing of speech sounds can be attributed to a failure of spike synchronization in the auditory nerve, a failure of compensatory plasticity in the central auditory pathway, or some combination thereof.

**Figure 6:**
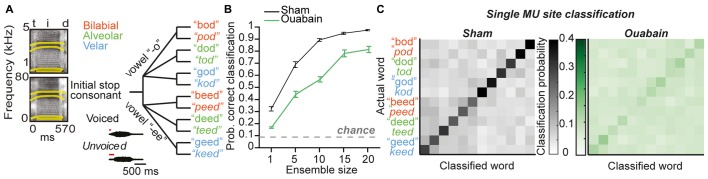
**Profound impairment in speech token classification in the MGB in ouabain treated mice, even after correcting for threshold shift and increasing ensemble size.** (**A**, left) Spectrograms of the speech token “teed” in original form (top) and resynthesized for mouse hearing (bottom). Formant frequencies are indicated by thick yellow lines. (**A**, right). Schematic of speech tokens arranged according to phonetic similarity. Acoustic properties that vary across speech tokens are indicated in insets. **(B)** Mean probability of correct classification of speech tokens using a 10 ms bin width across ensembles ranging from 1 to 20 multi-unit sites. Ouabain-treated animals show significant impairment, even though speech tokens were presented 20 dB above threshold for all sites. The probability of assigning the correct speech token identity by chance is indicated by the dashed gray line. **(C)** Mean confusion matrices from decoding single multi-unit PSTH patterns in sham (left) and ouabain (right) conditions. Axes are arranged according to schematic in **(B)**.

To investigate the neural basis of disrupted speech processing after auditory nerve damage, we recorded a human speaker producing 12 consonant-vowel-consonant combinations in a quiet background, and re-synthesized these speech tokens within the mouse hearing range with a method that preserves the spectrotemporal envelope structure of the original source material (see “Materials and Methods” Section, Figure 6A, left). A schematic showing both the acoustic differences between the categories of tokens varying across vowels, place of articulation, and voice onset timing (insets) as well as a tree plot showing the similarity of the tokens to each other (Figure 6A, right) demonstrates that the key differences between the initial stop consonants in each token occur within a time window of less than 50 ms. PSTHs taken in response to speech tokens presented at 20 dB above threshold were again binned at 10 ms and average classification accuracy was measured from either individual sites or ensembles of 5–20 sites (Figure 6B). Speech token classification was significantly impaired 30 days after cochlear denervation, even with larger ensembles that ensured almost 100% accuracy in the sham condition (*F*_(1)_ = 68.01, *p* < 0.0001, one-way ANOVA, Figure 6C, left and right). These findings highlight the impact of ouabain treatment on the representation of broadband, temporally modulated stimuli, and underscore the shortcomings of gain enhancement, threshold correction, resilient synchronization and population coding to fully compensate for profound cochlear denervation.

## Discussion

In this study, we confirmed that applying ouabain to the cochlear round window virtually eliminates the ABR while sparing cochlear transduction and outer hair cell-based amplification (Figure 1). We further showed that robust sound-evoked activity persists in a minority of MGB units recorded from mice with no measurable ABR (Figure 2) and that this persistent activity was sufficient to support frequency receptive fields (Figure 4) and classify sound intensities with comparable accuracy to control recordings at all but the lowest sound levels (Figure 3). Spike synchronization to broadband pulse trains was only modestly reduced, yet the overall classification accuracy of temporal pulse rate (Figure 5), and speech token identity (Figure 6) was greatly impaired.

**Figure 7:**
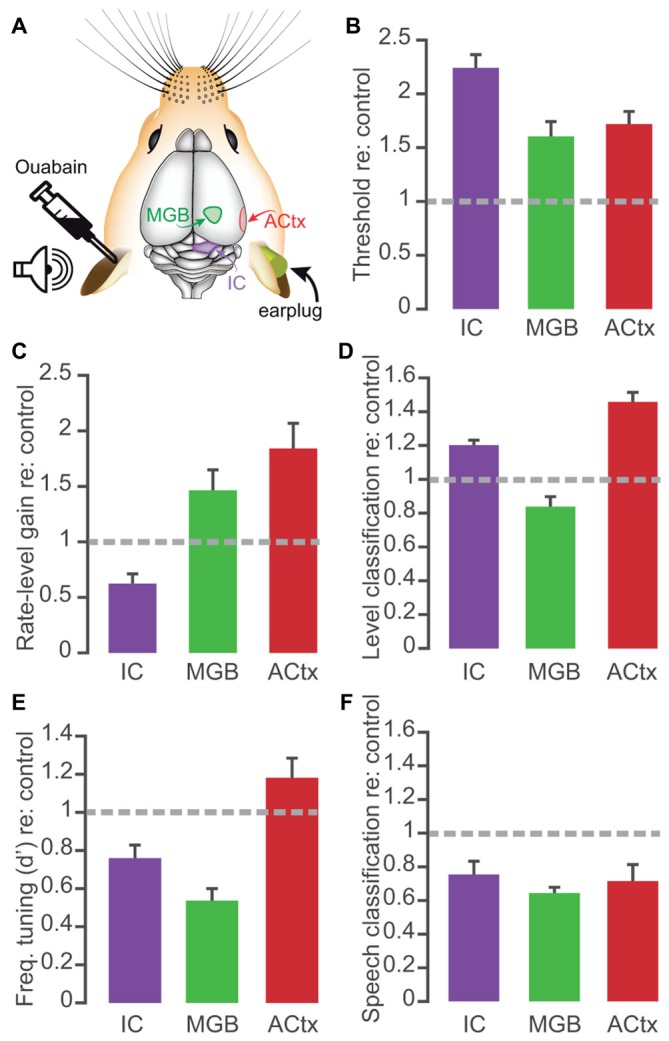
**Comparison of main effects of 30 days of recovery from ouabain treatment in the midbrain, thalamus and cortex. (A)** Schematic of color-coded recording locations, contralateral to the ouabain- or sham- treated ear. Auditory cortex (ACtx) and inferior colliculus (IC) recording results taken from Chambers et al. (2016). **(B–F)** Bar plots represent the mean ± SEM of rate-level threshold **(B)**, gain in spikes/s per 5 dB step **(C)**, probability of accurate level classification with a 10-ms PSTH bin size **(D)**, FRA d-prime value **(E)**, and probability of accurate speech token classification **(F)**. All values represent those taken at 30 days after ouabain treatment, normalized to the mean value from control recordings from the matched brain structure. For classification probability, mean values reflect the mean performance at each sound level or speech token, across the population of recording sites.

In comparison with our earlier report of central gain enhancement after unilateral ouabain treatment, the degree of compensatory plasticity in the thalamus is more modest overall than that observed than ACtx and in some cases, even IC (Figure 7). Although rate-level threshold shifts and gain in were perhaps more comparable to ACtx (Figures 7B,C), frequency tuning quality was most adversely affected in the MGB (Figure 7E). The thalamus showed a particularly weak recovery from ouabain treatment when sites were tested with the PSTH-based classifier, for both sound pressure levels (Figure 7D) and speech tokens (Figure 7F). Meanwhile the ACtx displayed enhancement via compensatory plasticity more reliably than the other two brain areas, providing strong evidence that the degree of plasticity observed in ACtx is not simply inherited from subcortical brain regions. However, technical caveats preclude a more detailed, statistical comparison of the datasets, including the fact that our earlier recordings were made from implanted electrodes in unrestrained mice as compared to the acute recordings made here from head-fixed mice.

One explanation for the variable degree of compensatory plasticity across the central auditory neuroaxis may be the difference in organization of inhibitory GABAergic circuits. Though the precise mechanisms for central gain adjustments after hearing loss likely reflect a combination of homeostatic and network changes that have yet to be fully elucidated in this context, most work points toward the central importance of disinhibition via reduced GABA tone (Dong et al., 2010; Lu et al., 2011; Kotak et al., 2013; Li et al., 2014; Nahmani and Turrigiano, 2014). In this regard, it is probably significant that the organization of GABA circuits in the rodent MGB is fundamentally different than cortex and is also fundamentally different from that of the MGB in carnivores and primates. In the ACtx, all inhibition is local; there are no feedforward GABAergic projections from the dorsal thalamus to primary sensory areas (Jones, 2007). In the rodent MGB, all inhibition is remote; there are virtually no GABA-synthesizing interneurons in the MGB (Ito et al., 2011; Hackett et al., 2015). In this regard, the rodent MGB differs from the MGB of other species, such as the cat, but also from the neighboring lateral geniculate nucleus, both of which have GABAergic interneurons (Arcelli et al., 1996; Winer and Larue, 1996). Synaptic inhibition in the MGB arrives via feedforward GABA projections from the IC (Winer et al., 1996; Peruzzi et al., 1997; Ito et al., 2011; Rudiger et al., 2013; Mellott et al., 2014) or from the reticular nucleus of the thalamus (Rouiller et al., 1985; Bartlett and Smith, 1999; Zhang et al., 2008), which makes precise topographic connections with sensory thalamic nuclei (Lam and Sherman, 2007; Cotillon-Williams et al., 2008).

GABA tone modulates stimulus processing in the MGB by, for example, shaping carrier frequency tuning functions and envelope modulation rate transfer functions (Cotillon-Williams et al., 2008; Cai and Caspary, 2015). Whereas many studies have linked sensorineural hearing loss to changes in GABA receptors that mediate rapid inhibitory synaptic currents in ACtx (Kotak et al., 2005, 2008; Scholl and Wehr, 2008) and IC (Mossop et al., 2000; Dong et al., 2009, 2010), the association between sensorineural hearing loss and synaptic inhibition in the MGB are less straightforward. Unlike the ACtx and IC, GABA currents in thalamic neurons are mainly trafficked through extra-synaptic receptors that impose tonic inhibition (Belelli et al., 2005; Cope et al., 2005; Herd et al., 2013). A recent study found that sensorineural hearing loss induced by noise exposure had no effect on fast synaptic inhibition mediated by GABA_A_ receptors in the MGB, but rather lead to an overall increase in tonic inhibition mediated by extrasynaptic GABA receptors (Sametsky et al., 2015). Elevated tonic inhibition shifted the firing mode of MGB neurons and may have disrupted the low-frequency electrical rhythms generated by the interplay of MGB, reticular nucleus and ACtx neurons, but evidently the effects of sensorineural hearing loss on MGB units is more complicated than the comparatively straightforward synaptic disinhibition described in the IC and ACtx. These key differences in the molecular pharmacology and circuit connectivity of thalamic GABA circuits may explain why central compensatory plasticity was more limited overall in the MGB than in ACtx in our own work and in previous studies of unilateral hearing loss (Hutson et al., 2008).

Although the compensatory plasticity at the level of the thalamus was not as large as observed elsewhere in the pathway, the auditory coding capabilities of MGB units after profound auditory denervation still far exceeded what would be expected solely from the ABR wave 1 data (Figure 1). Clinicians and researchers frequently use the ABR as a proxy for hearing status, even though sound perception is a psychological process that arises from spiking activity at higher stages of auditory processing. Auditory neuropathy is the exception that proves the rule, as basic audibility and sound-evoked cortical activity can be normal in ouabain-treated mice and patients on the auditory neuropathy spectrum, despite the virtual absence of an ABR or brainstem acoustic reflexes (Starr et al., 1996; Kraus et al., 2000; Zeng et al., 2005a; Lobarinas et al., 2013; Chambers et al., 2016). How could all measures of brainstem activity indicate profound hearing loss yet measures of forebrain activity and perception indicate normative audibility of simple stimuli, such as pure tones? One possibility is that diminished afferent signals coursing up the auditory nerve and brainstem are too weak to drive brainstem reflex pathways or the neural generators of the ABR, but these sensory traces are amplified at higher stages of central processing by hyper-excitable circuits.

Neurons at higher stations of sensory processing have a tremendous capacity to compensate for abrupt shifts in afferent activity levels and maintain invariant, stable sensory representations by tapping into homeostatic mechanisms that adjust intrinsic excitability (Desai et al., 1999), upward and downward scaling of sensitivity to glutamatergic and GABAergic neurotransmission (Watt and Desai, 2010; Turrigiano, 2012), and even activity-dependent oligodendrocyte myelination or modifications of the extracellular matrix (Bergles and Richardson, 2015). On the other hand, neurons at higher stages of auditory processing lack the requisite intrinsic and synaptic equipment needed for lossless encoding of stimulus fine structure or rapid envelope fluctuations that are characteristic of complex sounds, including speech. Correspondingly, decoding the identity of speech tokens from MGB (Figure 6) or ACtx spiking patterns remains greatly impaired after contralateral ouabain treatment, despite the persistent representation of rudimentary sound features, such as tones or noise tokens, which can be adequately encoded by variations in the overall firing rate (Chambers et al., 2016). The high-speed processing needed for temporal decoding of spectrotemporally complex stimuli likely remains beyond the reach of what can be recovered by central gain, and instead would require further recovery of brainstem circuits. Structured, intensive and early auditory training can improve temporal processing in the auditory brainstem (Strait et al., 2012; Parbery-Clark et al., 2013), as might cochlear therapies to regenerate or reconnect missing auditory nerve fibers (Wan et al., 2014; Suzuki et al., 2016). It remains to be seen whether these diverse approaches to auditory therapies could improve speech intelligibility in individuals with auditory neuropathy or other forms of hearing loss.

## Author Contributions

ARC collected preliminary data, analyzed data and wrote the manuscript, JJS collected and analyzed data, DBP designed the experiments and wrote the manuscript. All authors edited the manuscript.

## Funding

This work was supported by National Institutes of Health-R01DC009836, The Lauer Tinnitus Research Center and a Research Award from Autifony Therapeutics.

## Conflict of Interest Statement

The authors declare that the research was conducted in the absence of any commercial or financial relationships that could be construed as a potential conflict of interest.
